# Supplementary material for: Splice-Junction-Based Mapping of Alternative Isoforms in the Human Proteome
Source: Cell Rep. Author manuscript; Available in PMC 2020 Jan 15. (PMC6961840; doi:10.1016/j.celrep.2019.11.026)
Supplement: 2 [file NIHMS1546469-supplement-2.zip › DF1/PXD010154/Prostate/Prostate_15_TMEM259_SELLGLGSSMAGPPLLPR.pdf]

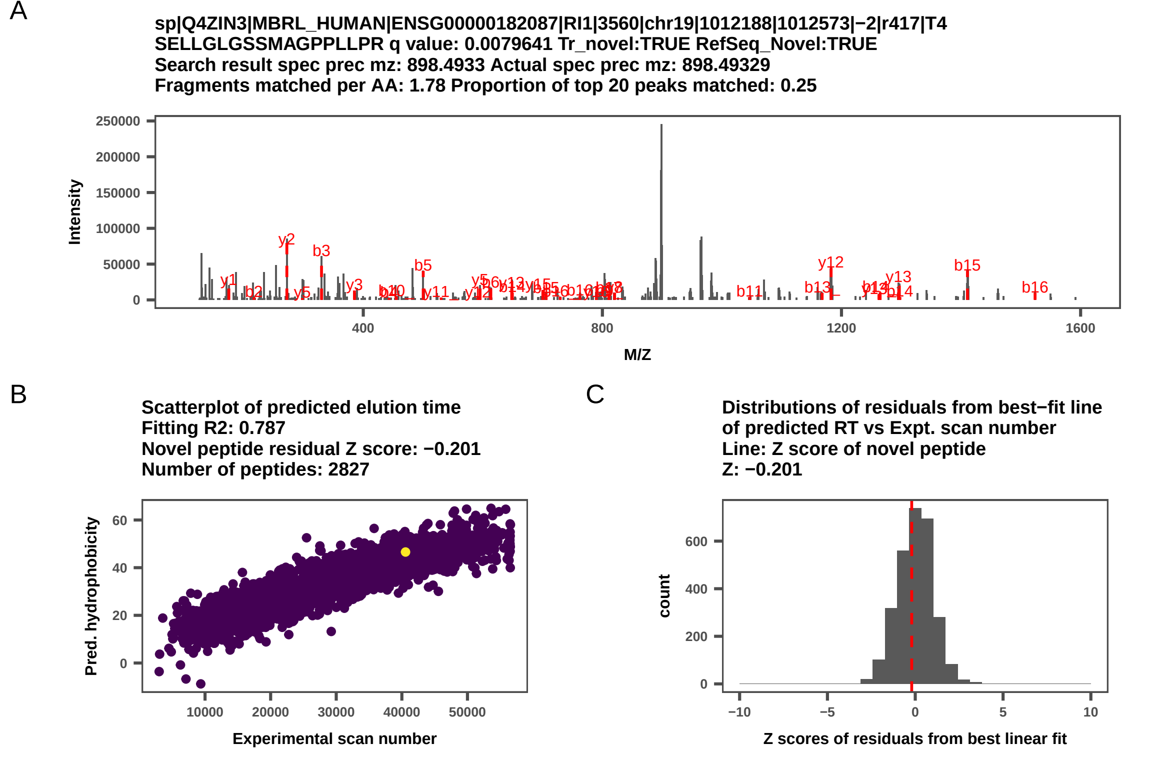
A
sp|Q4ZIN3|MBRL_HUMAN|ENSG00000182087|RI1|3560|chr19|1012188|1012573|−2|r417|T4
SELLGLGSSMAGPPLLPR q value: 0.0079641 Tr_novel:TRUE RefSeq_Novel:TRUE
Search result spec prec mz: 898.4933 Actual spec prec mz: 898.49329
Fragments matched per AA: 1.78 Proportion of top 20 peaks matched: 0.25
Intensity
250000
200000
150000
100000
50000
0
y1
y2
b2
y5
b3
y3
b5
y11
y12
b11
b13
y13
b15
b16
400
800
1200
1600
M/Z
B
Scatterplot of predicted elution time
Fitting R2: 0.787
Novel peptide residual Z score: −0.201
Number of peptides: 2827
Pred. hydrophobicity
60
40
20
0
10000
20000
30000
40000
50000
Experimental scan number
C
Distributions of residuals from best−fit line
of predicted RT vs Expt. scan number
Line: Z score of novel peptide
Z: −0.201
count
600
400
200
0
−10
−5
0
5
10
Z scores of residuals from best linear fit
